# Obtaining informed consent for genomics research in Africa: analysis of H3Africa consent documents

**DOI:** 10.1136/medethics-2015-102796

**Published:** 2015-12-07

**Authors:** Nchangwi Syntia Munung, Patricia Marshall, Megan Campbell, Katherine Littler, Francis Masiye, Odile Ouwe-Missi-Oukem-Boyer, Janet Seeley, D J Stein, Paulina Tindana, Jantina de Vries

**Affiliations:** 1 Department of Medicine, University of Cape Town, Cape Town, South Africa; 2 Center for Genetic Research Ethics and Law Department of Bioethics, School of Medicine, Case Western Reserve University, Cleaveland, Ohio, USA; 3 Department of Psychiatry and Mental Health, University of Cape Town, Cape Town, South Africa; 4 Wellcome Trust, London, UK; 5 Centre de Recherche Médicale et Sanitaire (CERMES), Niamey, Niger; 6 Cameroon Bioethics Initiative (CAMBIN), Yaounde, Cameroon; 7 MRC/UVRI Uganda Research Unit on AIDS, Entebbe, Uganda; 8 MRC Unit on Anxiety & Stress Disorders Department of Psychiatry and Mental Health, University of Cape Town, Cape Town, South Africa; 9 Navrongo Health Research Centre, Ghana Health Service, Navrongo, Ghana

**Keywords:** Informed Consent

## Abstract

**Background:**

The rise in genomic and biobanking research worldwide has led to the development of different informed consent models for use in such research. This study analyses consent documents used by investigators in the H3Africa (Human Heredity and Health in Africa) Consortium.

**Methods:**

A qualitative method for text analysis was used to analyse consent documents used in the collection of samples and data in H3Africa projects. Thematic domains included type of consent model, explanations of genetics/genomics, data sharing and feedback of test results.

**Results:**

Informed consent documents for 13 of the 19 H3Africa projects were analysed. Seven projects used broad consent, five projects used tiered consent and one used specific consent. Genetics was mostly explained in terms of inherited characteristics, heredity and health, genes and disease causation, or disease susceptibility. Only one project made provisions for the feedback of individual genetic results.

**Conclusion:**

H3Africa research makes use of three consent models—specific, tiered and broad consent. We outlined different strategies used by H3Africa investigators to explain concepts in genomics to potential research participants. To further ensure that the decision to participate in genomic research is informed and meaningful, we recommend that innovative approaches to the informed consent process be developed, preferably in consultation with research participants, research ethics committees and researchers in Africa.

## Introduction

The global interest in genomic and biobanking research has led to an evolving understanding of appropriate consent models for use in these types of investigations.^1^ ^2^ Consent models range from specific consent for the collection and use of human biological samples and data in a particular project to broad and blanket consent for all future uses, with several options in between.^3–5^ Tassé *et al*^3^ identified the following consent models currently in use: (1) broad and blanket consent; (2) tiered consent with different options for sharing and secondary use; (3) presumed consent for sharing; (4) recontacting or reconsenting for sharing; (5) waived consent; and (6) no consent (because no data with identifiers is used). In addition, some projects are exploring possibilities for dynamic consent, where research participants can provide consent on an ongoing basis using social media.^6^ ^7^

Most analyses of consent forms used in biobanking and genomic research^8^ ^9^ have focused on research taking place in Europe and North America. While there is now a small literature on consent for biobanking and genomics research in resource-limited locations, including African settings,^10–16^ many questions remain. For example, there are few data on the use of broad consent for health research in Africa including how key concepts in genetic and genomic research such as data and sample sharing, biobanking and reuse of samples collected as part of research are explained to research participants.

The Human Heredity and Health in Africa (H3Africa) Consortium is a collection of research and infrastructure projects seeking to apply genomics methodology to diseases affecting African people.^17^ Currently, H3Africa involves 26 funded projects: 15 genomics research projects, 4 biobanking projects, 6 Ethical, Legal and Social Implications projects and a pan-African bioinformatics network, H3ABioNet. Most of the genomics research projects involve several research sites across Africa. In 2014, the H3Africa Consortium developed guidelines for informed consent, which also contain template text for use in the development of project-specific consent documents (http://www.h3africa.org). These guidelines are not prescriptive and H3Africa researchers determine the most appropriate consent model considering the needs of their study population as well as their country-specific ethical and legal norms.

The development of H3Africa has prompted African researchers to grapple with the complexities around informed consent for genomics and biobanking research. The purpose of this paper is: (1) to describe how complex concepts in genomics are explained in consent documents used by H3Africa investigators; and (2) to explore consent models that are currently used in H3Africa projects.

## Methods

We sourced informed consent documents used in H3Africa projects. We contacted principal investigators (PIs) of the 15 genomics projects and the 4 biobanking projects. PIs were contacted via email and asked for copies of informed consent documents and supporting materials used for participant recruitment in their H3Africa projects. Documents were imported into NVivo V.10 software^18^ and coded. Where consent documents for different research sites differed substantially, we coded each source separately. Where there were minor variations in the names of places and people, we coded only one of the documents.

Two researchers (NSM and JdV) conducted the data analysis. Initial coding was performed to identify thematic domains.^19^ This was followed by a systematic review of these domains to ensure content validity. A coding scheme was developed which included the following items: (1) consent model (2) explanations of genetics/genomics; (3) explanations of data and sample sharing; (4) feedback of results and (5) H3Africa policies.^20^ ^21^ The application of codes was discussed and when necessary, content was recoded. Both researchers could read English and French allowing for all forms to be analysed in the original language. Some of these consent forms had been translated into other languages (Xhosa, Afrikaans, Swahili, Amaharic, Chichiwa, Chitumbuka and Luganda) but we did not include any of these translated versions in the current analysis. The initial project idea, preliminary findings and drafts of the manuscript were presented to and discussed with members of the H3Africa Working Group on Ethics and Regulatory Issues.

## Results

Of the 19 H3Africa genomics research studies and biobanking projects currently taking place, we received documents for 13 projects (12 research projects and 1 biobanking project). Most of the projects for which we received forms were enrolling participants in multiple sites, often across different African countries. One of these projects did not involve the collection of human biological samples per se, but of parasite samples from the human body. Three PIs informed us that they were not collecting (human) samples in their project. We did not receive a response from three other PIs. Together, the projects for which we analysed documents were engaged in sample collection in 22 countries across multiple sites. Most projects used the same consent documents in all sites, with minor variations in the names of places or people involved in enrolment. Only one project used documents that differed across study sites, with regards to the data and sample sharing descriptions. In total, 41 consent documents were collected. Of these, 3 were in French and 38 in English.

The length of the information sheets ranged from 2 to 11 pages, with an average length of 6.5 pages. Four projects had separate information sheets for different aspects of the study. For example, one project separated information about the main trait association study from information about the population genomic study, while another project separated information about sample sharing from the main study description. Where this was the case, we grouped the various information sheets together and analysed all information shared with participants.

### Consent models used by H3Africa projects

Of the 13 projects, 5 used a tiered consent model, 7 used a broad consent model and 1 used a specific consent model. One group began with a tiered consent model but moved to a broad consent model for pragmatic reasons. The project focusing on pathogen genomics did not mention data or sample sharing and only gave the option of consenting to be part of the study (specific consent). Of the four projects using tiered consent, two offered participants a choice between either sample destruction or depositing of samples in a biobank. The other two projects offered an additional choice between sharing for research in a disease-related field, or for ‘all’ future research.

### Explanation of genetics/genomics

We identified five strategies used by researchers to explain genetics and genomics to research participants. Explanations focused on heredity, heredity and health, genes and disease causation, disease susceptibility and progression, and heredity and phenotype (see table 1). Most of the 13 projects used a blend of these five different strategies at different locations in the consent documents, with 5 out of 13 blending two of these strategies. Two projects used one strategy, while three projects drew on three or more strategies. One project did not explain much about genetics but focused on explaining the disease under investigation.

**Table 1:** Strategies for explaining genetics/genomics in consent documents used in H3Africa studies

Defining genetics/ genomics	Common examples taken from the consent documents
Heredity (7 projects)	DNA is the code that you inherit from your parents and that you pass on to your children. This information may also be passed on from parent to child. This kind of information is passed from the father and the mother to their children and on to their grandchildren, in other words, from one generation to the next.
Heredity and health (3 projects)	Some illnesses are passed down in families because our DNA comes from our parents. To understand how inherited differences (traits that we get from our parents) influence our health. If an inherited change gives the person a health advantage, then people with that change will be more likely to survive and pass the change on to their children.
Genes and disease causation (5 projects)	Also, some, but not all, sicknesses can be caused by problems with DNA. Studying genes along with health information will help the researchers better understand what causes certain diseases. Aider à trouver le « gène » précis à l'origine du trouble médical dans votre famille (translation: Help to find the precise ‘gene’ that lies at the origin of the medical issues in your family’). We compare the DNA of the two groups so that we can see if there is any problem with the DNA causing the sicknesses.
Disease susceptibility and progression (3 projects)	To discover new genes, or new patterns in the way genes are used, that may help understand reasons for how quickly disease X progresses. Examine genes in people with disease X to help understand why some people develop their diseases faster than others. Some of these genes may prevent us from getting sick in the first place. Some other genes may be one of the reasons we get sick when others do not. Comment les changements au niveau du gène peuvent être responsables de vos symptômes (translation: How gene-level changes could be responsible for your symptoms).
Heredity and phenotype (physical traits) (4 projects)	These ‘genes’ are present in all of us and are what make people in families look like each other, but different from others. For example, some families are taller or shorter than others. The genetic material helps to decide for instance how tall you will be, what your body shape will be. This kind of information is passed from parents to children (which is why family members often look like each other).

The most common strategy used to explain genetics/genomics was by pointing to family inheritance, or the way that particular ‘information’ is passed from parents to children (Row 1 in table 1). Seven projects used this strategy. Of these, three projects linked this explanation to a discussion of diseases and how they are often passed on between family members (Row 2 in table 1). A further two projects started their explanation of genetics with the passing on of diseases in families. Four projects also linked such explanations to observations of how physical traits (height, body shape) are inherited in families (Row 5 in table 1).

Three projects linked their explanation of genetics and genomics to disease susceptibility and progression by highlighting the role of genes in influencing how quickly someone may become ill with a particular disease, or how they may respond to treatment (Row 4 in table 1). Three projects used a more ‘scientific’ explanation in defining genes. For example, genes were defined as ‘molecular units of heredity’ that ‘hold information about how our bodies work’ or that ‘carry the instructions for your body's development and function’. These three projects did not explain genetics any further.

With regards to the source of genomic material, some projects simply referred to ‘blood’ whereas others explained that the genetic material can be found in all cells in the body or in body tissues. Others did not specify a location of the genetic material but simply talked about ‘the genetic material in the body’.

### Explanation of data and sample sharing

All but one of the consent forms that we reviewed included a statement about data sharing. Ten out of 13 included a description of what sample sharing entailed. Of the three projects that did not include this description, two did not anticipate the need for sample sharing, while the third project collected only pathogen samples (taken from human samples). Of the projects that sought consent for sample and data sharing, most blended their descriptions of sample and data sharing into one.

In examining the forms, we identified four key elements associated with explanations of data and sample sharing: authorities deciding on reuse of samples, restrictions on secondary use, reasons for storing and definitions of biobanks (see table 2). Three projects specified that requests for secondary use would be reviewed by the ethics committees that approved the original study and one project specified that this task would fall to the Ministry of Health in the country where samples were collected. One project indicated that the funding agency would review requests for secondary use of samples, while four other projects indicated that this would be done by a special committee, a group of researchers or the biobank.

**Table 2:** Qualitative content analysis on different ways of explaining data and sample sharing

Data or sample sharing aspect	Description	Common examples taken from the consent documents
Authority deciding on reuse of samples	Research ethics committees that approved original study (4 projects)	These samples and related information may be used for other research studies in our country or abroad, pending ethical approval by our ethics committee
	Special committee, group of investigators or more broadly ‘permission from the biobank’ (4 projects)	A special committee will look at each request to study samples to find out what the researchers want to do and how they will protect your rights.
	Funding agency (1 project)	The control over samples you donate will be held by the funding agency.
	Ministry of Health (1 collaborating site in a project)	L'accès et l'utilisation de ces échantillons ne pourront se faire sans l'accord du Ministère de la Santé de notre pays (translation: Access and use of samples will have to be approved by the Ministry of Health)
Restrictions on secondary use	Only for ‘scientific’ or ‘medical’ research (5 projects)	Although the study you are being asked to participate in is related to (Disease X), other scientists may like to use your sample to study other diseases. They will need to agree only to use the data for scientific research.
	No restrictions (7 projects)	Investigators from all over the world can use these samples for their research; samples may be used to study other diseases.
	Specific diseases (1 project and 1 collaborating site in a project)	les échantillons vont être conservés en attendant leur utilisation par les chercheurs et projets de recherches associés à notre projet (translation: (the samples) will be stored for reuse by researchers and projects associated with our project)
Reasons for storing	To boost the power of studies and research (2 projects)	To do more powerful research, it is helpful for researchers to share information they get from studying human samples
	Because this is now best practice (4 projects)	It is now common that genetic information is shared with researchers around the world, for other research in the future
	Because it is the right thing to do (2 projects)	A goal of H3Africa is to create a way for investigators to share and learn from each other, especially within Africa. One of the best ways to do this is for scientists to share research information
Definitions of Biobanks	(7 projects)	The storage place also known as a biorepository is a collection of samples and health information from many people, stored for study. A sophisticated blood storage facility. Some of the samples may be stored as part of a big collection or ‘biobank’.‘A biobank is a place that stores samples and information so that researchers on this study and other scientists can use them in future unspecified research projects”

Five of the 13 projects mentioned a timeline for sample storage noting that samples would be stored either indefinitely (2 projects), for the study duration (1 project) or for 15 years (2 projects). However, the majority of projects (8) did not mention the length of storage. With regards to describing who may use the data, most forms were rather broad, indicating that ‘other researchers around the world’ could use the data for ‘other projects'. Two projects restricted the utility of the data to a particular disease or disease group, while four projects indicated that samples and data would only be used for ‘scientific research’ or ‘medical research’. Two projects detailed that samples and data could be used by private sector investigators.

We examined reasons given for storing, which corresponds to sharing samples and data across the projects and found that while three projects gave no reason for sample sharing, five main reasons were provided by the other projects. These included: best practice (four projects), the right thing to do (two projects), to facilitate future unspecified research (four projects), for the benefit of science and medicine (one project), and to boost the power of studies (two projects) (table 2).

In our assessment of how biobanks are explained in consent forms, we found that seven projects offered some description of what a biobank is, for instance that it is a ‘place where samples are stored’. In terms of the location of the biobanks, eight projects stated that this will be ‘somewhere on the African continent’, two projects detailed the specific (African and non-African) countries where samples will be stored, while three projects did not mention a location.

### Feedback of genetic and non-genetic results

In our analysis of feedback of study results, we separated genetic study results from other test results. Nine projects indicated that they would provide feedback on individual non-genetic test results to research participants, while four projects did not mention providing any feedback to participants. A variety of non-genetic results were described in the consent forms, including information about parasite density, blood pressure, blood sugar and lipids, and results of echocardiograms.

Regarding feedback of individual-level genetic study results, six projects did not mention whether they would return genetic research results, while five projects specified that no genetic research results would be returned. Reasons given for not returning genetic results included that it could take a long time before results would be known and that there is an incomplete understanding of the role of genes in disease causation. Two projects described the possibility that some results could be shared in the future, using non-committal phrases like ‘there is a small chance we may find something important. If this happens, we may contact you to find out if you would like to learn more’. One project indicated that participants could obtain the results of the genetic tests at their request. This project provided considerable detail about the types of findings that would be given to participants. It distinguished treatable medical conditions that have a clear genetic origin (which would be fed back), from conditions for which genetic predisposition is only a contributing factor (no feedback on these conditions would be provided to participants). All relevant findings would be verified in a diagnostic facility and feedback would be given by a study doctor and a genetic counsellor.

### H3Africa policies

Six of the 13 projects did not refer to the H3Africa Consortium in their consent forms. Of the other projects, most made minimal mention, describing for instance that ‘this study is part of the H3Africa project’, or ‘samples will be held in an H3Africa biobank’ but without further details. None of the projects referred to the H3Africa policy framework, which is not surprising as most projects developed their consent documents before the policy framework was developed.

## Discussion

In this article, which comprises the first comprehensive analysis of consent documents and models used in the recruitment of research participants for genomics and biobanking research in Africa, we have documented how H3Africa researchers explain key concepts of genomic research to study participants and the type of consent models used in H3Africa projects.

There are ongoing discussions about the appropriateness of using broad consent when recruiting research participants with low health-literacy in resource-poor settings.^22^ ^23^ Challenges relating to the use of broad consent models in Africa are multiple and include questions about research participant comprehension of concepts in genomic research, future use of samples collected as part of research and the possible risk of stigma or exploitation of study communities.^24^ ^25^ There is also a regulatory gap and limited legal and ethical guidance available in Africa to support a transition from specific to broad consent models.^26–28^ Taken together, these questions translate into considerable apprehension by African research ethics committees to approve research that makes use of broad consent. Despite these concerns, our study shows that most H3Africa projects adopted a broad consent model.

In H3Africa, broad consent for genomic and biobanking studies is currently mandated by funding requirements. For genomics research, the sharing of data for secondary use is now standard practice, and a requirement imposed by most of the large funding agencies.^29^ ^30^ Similarly, biobanking is only meaningful if samples are shared widely, and if consent is broad enough to allow for wide reuse of samples. The introduction of broad consent requirements in African health research may be good—for instance, if broad consent cannot be used for the recruitment of African research participants in genomics and biobanking research, then it is possible that Africa will be further excluded from genomics and biobanking research, thus not remedying the existing underrepresentation of African people in such research^31^ and preventing African populations from harnessing the potential health benefits of human genomics research^17^ ^29^—an outcome that would clearly be unjust.^32^ This is particularly true when there is emerging evidence from Africa that research participants are supportive of broad consent where this promotes health research and reduces global health inequality.^33^ ^34^

But at the same time, the requirement for broad consent in H3Africa research raises questions about the way in which ethical norms in Africa are formulated and evolve. Medical research in Africa is closely associated with concerns of imperialism and exploitation.^35^ ^36^ The imposition of non-African ethical standards on research in Africa has also long been a concern in global health ethics.^37^ ^38^ The real challenge at stake is to find a way to foster African deliberation on and adaptation of ethical norms introduced by novel scientific practices, without this process compromising potential benefit of African genomics research to patients. Within the context of H3Africa, the approach has been to foster ethical deliberation^32^ while also getting on with research—but whether this is indeed the best approach to fostering ethical debate remains to be seen.

Informed consent is one important strategy to avoid potential exploitation of research participants and protect their rights and well-being^39^—but only if the consent process is designed in a way that is culturally appropriate and understandable. Investigators examining consent to genomic research in African settings have identified a number of challenges in communicating study goals, methods and procedures.^12–14^ ^40^ This is compounded by difficulties in finding equivalent terminology to explain pertinent concepts in local languages.^12^ In our examination of consent documents, we found that researchers sought to find layperson explanations of some of the difficult concepts in genomics. We demonstrated that there was diversity across projects in the strategies used to explain key concepts of genomics research. However, consistent across many documents was reference to some notion of heredity, on its own or as linked to phenotype, disease causation or progression as a means of explaining genomics and genetics.

The need for sample and data sharing was explained by suggesting that this represents scientific best practice, that it facilitates future research and that it would benefit science and medicine. In terms of secondary use of samples, many projects suggested that this will be done by a local Research Ethics Committee (REC) and/or a ‘special committee'. This reflects the views of some African REC members that the reuse of samples collected as part of research has to be monitored by African RECs.^22^ ^23^ ^25^ The H3Africa policy framework however suggests that a Data and Biospecimen Access Committee should decide on sample and data access.^32^ This potential tension between the content of H3A consent forms and the policy framework will need to be addressed in the actual management of secondary sample and data access. This observation raises larger questions about the content of consent documents and how this is respected down the line.

The absence of information about the feedback of individual genetic study results in most forms—and a clear commitment to return genetic results in only one project—illustrates the fact that in Africa, there is little or no experience with or opportunity for integrating genetic findings into personal healthcare, as evidenced by the nearly complete absence of this topic in academic literature.^41^ The question of whether and how health related findings in genomic research should and could be returned to African research participants will require further empirical work. Arguably, the feedback of study findings could lead to an increase in the number of people that need to be followed up in the healthcare system, which in many African countries are already overburdened and struggling to cope with patients requiring acute care. The question is whether the addition of patients who do not yet manifest disease is morally acceptable.

One limitation of our analysis is that we focused on English and French language consent documents, and did not include the direct translations made into local African languages. We also did not consider how local language versions of these informed consent documents are used in practice. We did not include translations for a number of reasons, most importantly because of the immensity of the task involved. As we indicated, the forms we analysed are used in multiple research sites across 22 countries, and each project may translate consent documents into three or four languages. This would mean that we would have had to deal with over 100 translations—the analysis of which would have had to be done by people able to read the languages involved. Although important, we also think that this was outside of the scope of the project. However, it is of key importance that our analysis be accompanied by studies examining the use of linguistic and conceptually equivalent terminology in local African languages to explain pertinent concepts in genomics, and by empirical studies examining the broader consent processes, including participant comprehension.
